# Adipose triglyceride lipase acts on neutrophil lipid droplets to regulate substrate availability for lipid mediator synthesis

**DOI:** 10.1189/jlb.3A0515-206R

**Published:** 2015-06-24

**Authors:** Stefanie Schlager, Madeleine Goeritzer, Katharina Jandl, Robert Frei, Nemanja Vujic, Dagmar Kolb, Heimo Strohmaier, Juliane Dorow, Thomas O. Eichmann, Angelika Rosenberger, Albert Wölfler, Achim Lass, Erin E. Kershaw, Uta Ceglarek, Andrea Dichlberger, Akos Heinemann, Dagmar Kratky

**Affiliations:** Institutes of *Molecular Biology and Biochemistry and ^†^Experimental and Clinical Pharmacology, ^‡^Center for Medical Research, and ^#^Division of Hematology, Medical University of Graz, Graz, Austria; ^§^Institute of Laboratory Medicine, Clinical Chemistry and Molecular Diagnostics, University Hospital Leipzig, Leipzig, Germany; ^¶^LIFE–Leipzig Research Center for Civilization Diseases, University of Leipzig, Leipzig, Germany; ^‖^Institute of Molecular Biosciences, University of Graz, Graz, Austria; **Division of Endocrinology and Metabolism, Department of Medicine, University of Pittsburgh, Pittsburgh, Pennsylvania, USA; and ^††^Department of Anatomy, Faculty of Medicine, University of Helsinki, Helsinki, Finland

**Keywords:** inflammatory cells, arachidonic acid, eicosanoids, lipolysis

## Abstract

In humans, mutations in ATGL lead to TG accumulation in LDs of most tissues and cells, including peripheral blood leukocytes. This pathologic condition is called Jordans’ anomaly, in which functional consequences have not been investigated. In the present study, we tested the hypothesis that ATGL plays a role in leukocyte LD metabolism and immune cell function. Similar to humans with loss-of-function mutations in ATGL, we found that global and myeloid-specific Atgl^−/−^ mice exhibit Jordans’ anomaly with increased abundance of intracellular TG-rich LDs in neutrophil granulocytes. In a model of inflammatory peritonitis, lipid accumulation was also observed in monocytes and macrophages but not in eosinophils or lymphocytes. Neutrophils from Atgl^−/−^ mice showed enhanced immune responses in vitro, which were more prominent in cells from global compared with myeloid-specific Atgl^−/−^ mice. Mechanistically, ATGL^−/−^ as well as pharmacological inhibition of ATGL led to an impaired release of lipid mediators from neutrophils. These findings demonstrate that the release of lipid mediators is dependent on the liberation of precursor molecules from the TG-rich pool of LDs by ATGL. Our data provide mechanistic insights into Jordans’ anomaly in neutrophils and suggest that ATGL is a potent regulator of immune cell function and inflammatory diseases.

## Abstract (precis)

Lipid mediator release depends on the hydrolysis of triglyceride-rich lipid droplets mediated by ATGL, a potent regulator of inflammatory diseases.

## Introduction

NLSD describes a group of rare, autosomal-recessive disorders of lipid metabolism with systemic deposition of neutral lipids in multiple tissues. The disease was discovered initially in 1953 by Jordans [1], who reported a case of progressive muscular dystrophy accompanied by fat-containing vacuoles in leukocytes, which, since then, has been termed Jordans’ anomaly. Lipid-laden leukocytes were confirmed in further clinical reports of NLSD [2–4]. Given the consistent presence of Jordans’ anomaly on an otherwise heterogeneous background of clinical phenotypes (e.g., variable degrees of ichthyosis, liver steatosis, hepatosplenomegaly, skeletal and cardiac myopathy, hearing loss, growth, and mental retardation) [5], Jordans’ anomaly was proposed as hallmark criterion for the diagnosis of NLSD [6]. In 2001 and 2007, mutations in CGI-58 (also termed α/β hydrolase domain-containing 5) and ATGL (also termed desnutrin or PNPLA2) were linked to NLSD with ichthyosis and NLSDM, respectively [7, 8]. ATGL and CGI-58 are key regulators of lipolysis. The catabolism of TG-rich intracellular LDs takes place predominantly in adipose tissue. It is initiated by ATGL, followed by the action of HSL and MGL, and it largely depends on the presence of the ATGL coactivator CGI-58 [9]. As LDs have been recognized as functional and dynamic organelles beyond lipid storage depots for metabolic fuel, novel implications of LDs and their associated proteins in various cell types are steadily emerging. LDs have been linked to a variety of biologic functions, such as cellular regulation of lipid homeostasis and signaling, protein folding, storage and degradation, infection, and immunity (reviewed in refs. [10–12]). Several human diseases (e.g., inflammatory arthritis, respiratory distress syndrome, and atherosclerosis) are associated with LD accumulation in various types of leukocytes, implicating LDs as critical regulators of inflammation. Significant inflammation-relevant functions that have been associated with LDs (often termed lipid bodies in the context of inflammation) include the regulation of host/pathogen interactions [13], virus assembly [14], nutrient source for intracellular pathogens [15], and functions in antigen cross-presentation [16]. Numerous studies have provided substantial evidence that LDs serve as major organelles for the generation of AA-derived lipid mediators (reviewed in ref. [17]).

In cells of the myeloblastic lineage (e.g., neutrophils, eosinophils, basophils, mast cells), TGs are the major lipid class of the LD core [18–20]. However, the function of lipolytic enzymes that use this TG pool in cells of the innate or adaptive immune system is largely unexplored. Here, we investigated the role of ATGL in leukocyte LD biology and immune cell function by examining neutrophils lacking ATGL. Our study shows that similar to humans with defective ATGL, Atgl^−/−^ mice exhibit Jordans’ anomaly. In addition, ATGL^−/−^ leads to alterations in immune cell function and a marked reduction in lipid mediator generation. To our knowledge, this is the first report providing detailed information about the functional consequences of Jordans’ anomaly caused by ATGL^−/−^.

## MATERIALS AND METHODS

### Animals

Global Atgl^−/−^ mice were generated, as described elsewhere [21]. Mice carrying a LoxP-modified Atgl allele (B6.129-Pnpla2^tm1Eek^ mice; backcrossed onto C57BL/6 × N3; herein designated as Atgl^flox^ mice) were generated as described [22]. Mice with a targeted deletion of Atgl in myeloid cells (myeloid Atgl^−/−^ mice) were obtained by crossing Atgl^flox/flox^ mice with transgenic mice that express Cre recombinase under the control of the murine M lysozyme promoter (LysMCre; C57BL/6 background; provided by Dr. Thomas Ruelicke, University of Veterinary Medicine Vienna, Austria). Atgl^flox/flox^ mice were used as controls (myeloid WT). Mice were kept on a standard chow diet (4% fat and 19% protein; Altromin Spezialfutter GmbH & Co., Lage, Germany) and water ad libidum on a regular light-dark cycle (12 h light, 12 h dark). Mice were genotyped by use of the following primers: Atgl-forward 5′-AGAGAGAGAAGCTGAAGCCTG-3′, Atgl-reverse 5′-GCCAGCGAATGAGATGTTCC-3′; Atgl^flox/flox^-forward 5′-CGGTGAGGGTGGGGAACGGAGTC-3′, Atgl^flox/flox^-reverse 5′-CAGGGGGCCAGGCGGTCAGA-3′; Cre-mutated 5′-CCCAGAAATGCCAGATTACG-3′; Cre-common 5′-CTTGGGCTGCCAGAATTTCTC-3′; Cre-WT 5′-TTACAGTCGGCCAGGCTGAC-3′. Animal protocols were approved by the Austrian Federal Ministry of Science, Research and Economy, Division of Genetic Engineering and Animal Experiments (Vienna, Austria; BMWF-66.010/0085-II/3b/2013, BMWF-66.010/0081-II/3b/2013, BMWFW-66.010/0076-WF/II/3b/2014).

### Isolation of neutrophils

Neutrophils were isolated from peritoneal exudate or bone marrow. Cells of peritoneal exudate were collected 1 d after intraperitoneal injection of 2.5 ml 3% thioglycolate by rinsing the peritoneum with 10 ml HBSS containing 1 mM EDTA. For bone marrow isolation, tibia and femur from both hind legs were collected, flushed with HBSS-EDTA, and pushed through a cell strainer to disperse cell clumps. The cell suspension was centrifuged (400 *g*, 10 min, 4°C), and neutrophils were purified by use of a Percoll gradient, as described [23]. In brief, cell suspensions were layered on top of a 3-layer Percoll gradient of 78, 69, and 52% Percoll and centrifuged (1500 *g*, 30 min, room temperature) with brakes off. Neutrophils from the 69–78% interphase were harvested and counted. After washing with HBSS-EDTA containing 1% BSA, cells were suspended in HEPES buffer plus 9 mM glucose and used immediately for further experiments.

### Isolation of inflammatory cells for quantitative real-time PCR

Cells were flow sorted by use of a BD FACSAria III cell sorter (BD Biosciences, San Jose, CA, USA). Peritoneal lavages from WT mice, 2 d after thioglycolate injection, were used to sort eosinophils, neutrophils, monocytes, and macrophages. B and T lymphocytes were sorted from peripheral blood after RBC lysis in ammonium-chloride-potassium lysis buffer (150 mM NH_4_Cl, 10 mM KHCO_3_, 0.1 mM Na_2_ EDTA, pH 7.2). Total RNA from cells was extracted by use of TriFast reagent, according to the manufacturer’s protocol (Peqlab, Erlangen, Germany). Total RNA (1 µg) was reverse transcribed by use of the High-Capacity cDNA Reverse Transcription Kit (Applied Biosystems, Carlsbad, CA, USA). Quantitative real-time PCR was performed on a LightCycler 480 (Roche Diagnostics, Rotkreuz, Switzerland) by use of the QuantiFast SYBR Green PCR kit (Qiagen, Hilden, Germany). For data normalization, *GAPDH* was used as an endogenous control, and the relative units for gene expression were calculated by use of the 2^−ΔΔCt^ method. The following primer sequences were used for quantitative PCR: *GAPDH*-forward 5′-AGGTCGGTGTGAACGGATTTG-3′, *GAPDH*-reverse 5′-GGGGTCGTTGATGGCAACA-3′; *ATGL*-forward 5′-GCCACTCACATCTACGGAGC-3′, *ATGL*-reverse 5′-GACAGCCACGGATGGTGTTC-3′; *HSL*-forward 5′-GCTGGTGACACTCGCAGAAG-3′, *HSL*-reverse 5′-TGGCTGGTGTCTCTGTGTCC-3′; *MGL*- forward 5′-GCCACTCACATCTACGGAGC-3′, *MGL*-reverse 5′-GACAGCCACGGATGGTGTTC-3′.

### Immunophenotyping and lipid staining of peripheral blood and peritoneal and bone marrow cells

Peripheral blood cells were collected by retrobulbar puncture from isoflurane-anesthetized mice. Peritoneal lavage cells were collected as mentioned above. After RBC lysis, remaining cells were fixed with 10% methanol-free formalin for 10 min at 4°C and blocked with PBS containing 10% FCS for 10 min at 4°C. After the incubation, cells were stained in PBS containing 3% FCS for 20 min at 4°C with the following antibodies against surface markers: CD11b-Alexa Fluor 647, SiglecF-PE, and Gr-1-APC-Cy7 (all purchased from BD Biosciences); CD115-PE-Cy7, CD19-eFluor 605, and CD3-eFluor 450 antibodies (all purchased from eBioscience, San Diego, CA, USA) for peripheral blood cells; F4/80-eFluor 450 (eBioscience); and SiglecF-PE, Gr-1-PerCP-Cy5.5, CD19-PE-Cy7, and CD3-APC antibodies (all purchased from BD Biosciences) for peritoneal cells. All antibodies were titrated before use. The frequencies of specific cell types were calculated as the percentage of living cells. Intracellular LDs were quantified by staining with the fluorescent dye BODIPY 493/503 (1 µg/ml; Life Technologies, Carlsbad, CA, USA). After 10 min of incubation at 4°C, cells were washed, resuspended in PBS, and analyzed (1 × 10^5^ cells/measurement) by use of an LSR II flow cytometer (BD Biosciences). Data were acquired by use of DIVA 6.1.2 software (BD Biosciences), and the analysis was performed by use of FlowJo (Tree Star, San Carlos, CA, USA). For bone marrow analysis, femurs and tibias were collected, and the marrow was flushed out of the bones with HBSS-EDTA. Cell pellets were resuspended in 200 µl antibody cocktail and incubated at room temperature for 10 min. For LD staining, BODIPY 493/503 was added to the cells for another 10 min. Finally, cells were washed in buffer (PBS, 0.5% BSA, 0.025% NaN_3_) and analyzed immediately. A forward-/side-scatter gate excluded cell debris and remaining RBCs, and dead cells were excluded by 7-aminoactinomycin D (BD Biosciences) uptake. LSK populations were defined as Lin^−^Sca-1^+^c-Kit^+^, GMPs as Lin^−^c-Kit^+^CD34^+^FcγRII/III^+^, MDPs as Lin^−^c-Kit^+/Int^CD115^+^Flt3^+^, and CLPs as Lin^−^c-Kit^lo^Sca-1^lo^IL-7Rα^+^. CD115-PE, CD117(c-Kit)-PE-Cy7, CD16/32(FcγRII/III)-eFluor 450, and Ly-6A/E(Sca-1)-PE-Cy7 antibodies were purchased from eBioscience. IL-7Rα(CD127)-brilliant violet and Flt3(CD135)-APC antibodies were purchased from BioLegend (San Diego, CA, USA) and CD117(c-Kit)-APC from BD Biosciences. The lineage antibody cocktail encompassed anti-Gr-1, anti-CD11b, anti-Ter119, anti-B220, and anti-CD3e (BD Biosciences).

### Transmission electron microscopy

Bone marrow cells were fixed in 2.5% glutaraldehyde (w/v) and 5% methanol-free formalin in 0.1 M phosphate buffer (pH 7.4) for 2 h at room temperature, postfixed in 2% osmium tetroxide (w/v) for 2 h, dehydrated in a graded series of ethanol, and embedded in a TAAB epoxy resin. Ultrathin sections (75 nm) were cut with a Leica UC6 Ultramicrotome (Leica Microsystems, Wetzlar, Germany) and stained with lead citrate for 5 min and with uranyl acetate for 15 min. Images were taken by use of a FEI Tecnai G2 20 transmission electron microscope (FEI, Eindhoven, Netherlands) with a Gatan Ultrascan 1000 charge-coupled device camera. Acceleration voltage was 120 kV.

### Western blotting analysis

Protein concentrations from cell lysates were determined by the Bio-Rad DC Protein Assay (Bio-Rad Laboratories, Hercules, CA, USA). Proteins were separated by SDS-PAGE under reducing conditions (25 mM DTT) and then transferred onto a nitrocellulose membrane (Hybond-C Extra; Amersham Biosciences, Piscataway, NJ, USA). Nonspecific binding sites were blocked by incubating the membrane with 5% nonfat dry milk in 1× TBST buffer (150 mM NaCl, 10 mM Tris, 0.1% Tween 20, pH 8) for 1 h at room temperature. Immunodetection was performed by use of mouse anti-ATGL (1:200), mouse anti-HSL (1:800; Cell Signaling Technology, Danvers, MA, USA), rabbit anti-MGL antibody (1:1000; kindly provided by Dr. Robert Zimmermann, University of Graz, Graz, Austria), and anti-mouse β-actin (1:10,000; Sigma-Aldrich, Steinheim, Germany). The HRP peroxidase-conjugated goat anti-rabbit (1:5000) and rabbit anti-mouse antibodies (1:1000; Dako, Glostrup, Denmark) were visualized by ECL detection (ECL Plus; Thermo Scientific, Rockford, IL, USA) by use of a ChemiDoc MP Imaging System (Bio-Rad Laboratories).

### Lipid analysis of peritoneal lavage

Peritoneal lavages were harvested from mice 1 d after intraperitoneal thioglycolate injection. Lipids from peritoneal exudates were extracted by use of the Folch method. After evaporation under constant nitrogen flow, 100 µl 1% Triton X-100 (dissolved in chloroform) was added, and lipids were dried again under a stream of nitrogen. Thereafter, samples were dissolved in 100 µl ddH_2_O for 15 min at 37°C. Aliquots were used for enzymatic measurements of TG, TC, and FC by use of kits, according to the manufacturer’s protocols (DiaSys, Holzheim, Germany). The readings were normalized to protein concentrations. To determine the composition of TG, PC, and phosphatidylethanolamine species, extracted lipids were dissolved in 2-propanol:chloroform:methanol (7:2:1 v:v:v) and analyzed by LC/ESI-MS, as described [24].

### Calcium flux and chemotaxis assays

Free [Ca^2+^]_i_ levels of bone marrow-derived neutrophils were analyzed in response to fMLP (Sigma-Aldrich), and KC (PeproTech, Rocky Hill, NJ, USA). Changes in [Ca^2+^]_i_ were detected by flow cytometry as an increase in fluorescence intensity of the Ca^2+^-sensitive dye Fluo-3 in the fluorescence 1 channel. Cells (1 × 10^7^) were incubated with 500 µl assay buffer (10 mM HEPES, 10 mM glucose, 0.1% BSA, pH 7.4) containing 2 µM Fluo-3 acetoxymethylester (Life Technologies) and 0.02% pluronic F-127 (Sigma-Aldrich) in the absence of Ca^2+^ and Mg^2+^ for 1 h at room temperature. Cells were washed with assay buffer and resuspended in assay buffer containing Ca^2+^ and Mg^2+^ to a final concentration of 0.3–0.5 × 10^6^ neutrophils/ml. Aliquots of 500 µl were used for each condition. Maximal Ca^2+^ responses were determined after adding various concentrations of fMLP and KC. Kinetics of Ca^2+^ flux were analyzed by use of FlowJo software (Tree Star).

For chemotaxis assays, bone marrow-derived neutrophils were suspended in assay buffer containing Ca^2+^ and Mg^2+^ (2 × 10^6^ cells/ml). Cell suspension (50 µl/well) were placed onto the top plate of an AP48 micro-Boyden chemotaxis chamber with a 5 µm pore-size polycarbonate filter (Neuro Probe, Gaithersburg, MD, USA). Cells were allowed to migrate toward 30 µl fMLP and KC in the bottom wells of the plate for 1 h at 37°C. The membrane was removed carefully, and migrated cells were enumerated by flow cytometric counting.

### FA uptake

Peritoneal neutrophils (1 d after thioglycolate injection) were resuspended in HBSS containing Ca^2+^, Mg^2+^, and 0.1% FA-free BSA. After 15 min, 5 µM BODIPY 500/510 C1, C12 (Life Technologies) was added for 10 min. Thereafter, cells were fixed with 10% methanol-free formalin for 10 min at 4°C and washed 2 times with HBSS-EDTA, stained with a neutrophil-specific marker (Ly6G-PE; BD Biosciences), and analyzed by flow cytometry.

### Phagocytosis and ROS measurement

Neutrophils were isolated from peritoneal lavages, 1 d after thioglycolate injection. After 1 h of preincubation in culture medium (DMEM, 4 mM glutamine, 1 mM pyruvate, 10% lipoprotein-deficient serum, 1% penicillin/streptomycin) in the absence of glucose for 1 h, neutrophils (1 × 10^6^) were incubated with 100 µl fluorescein-labeled *Escherichia coli* particles (Vybrant phagocytosis assay; Molecular Probes, Life Technologies) for 1 h at 37°C in the dark under gentle rotation. The suspension was removed, and 100 μl trypan blue was added for 1 min to quench extracellular fluorescein-labeled bacteria. Cells were washed with cold HBSS-EDTA and stained with a neutrophil-specific marker (Ly6G-PE; BD Biosciences), and the amount of phagocytosed *E. coli* was measured by flow cytometry. Data are expressed as geometric means of fluorescence intensity of the intracellular fluorescence detected. Intracellular ROS generation, during phagocytosis and after a 20 min exposure to KC (20 ng/ml) and fMLP (1 µM), was measured according to the manufacturer's protocol (Life Technologies).

### Inhibition of ATGL in vitro after FA loading

Peritoneal neutrophils were cultivated in culture medium containing 25 mM glucose, supplemented with 300 µM OA or AA (Sigma-Aldrich), complexed to BSA (OA-BSA, AA-BSA). FA:BSA complexes were prepared as described [25]. After an incubation period of 6 h in the absence or presence of the ATGL inhibitor Atglistatin (40 µM) and Hi (25 µM; kindly provided by Dr. Martina Schweiger, University of Graz), the medium was replaced by serum-free DMEM containing Atglistatin or Atglistatin/Hi. After 4 and 6 h of incubation, LDs were quantified by flow cytometry, as described above.

### Quantification of PUFAs and lipid mediators

Peritoneal neutrophils were preincubated in culture medium (containing 25 mM glucose), with or without 40 µM Atglistatin, for 6 h. Thereafter, the medium was replaced by HBSS containing Ca^2+^ and Mg^2+^ in the absence or presence of the Ca^2+^ ionophore A23187 (0.5 µM; Sigma-Aldrich) for 1 h. Then, cells were placed on ice, transferred into tubes, and centrifuged at 400 *g* for 10 min at 4°C. Supernatants were immediately frozen in liquid N_2_ and stored at −80°C (maximum 1 wk) until analysis. To determine PUFA and lipid mediator concentrations in peritoneal lavage fluids, the peritoneum was rinsed with 3 ml HBSS-EDTA. The cell-free exudate was snap frozen in liquid N_2_ and stored at −80°C. A QTRAP 5500 mass spectrometer (AB Sciex, Darmstadt, Germany), operating in negative ESI mode, was applied for LC-MS/MS analysis by use of 200 µl cell supernatant or peritoneal lavage fluid, as described previously [26].

### Statistical analysis

Statistical analyses were performed by use of GraphPad Prism 5.0 software (GraphPad Software, La Jolla, CA, USA). Significance was calculated by Student’s *t* test or ANOVA, followed by Bonferroni correction. Data are shown as means + sem. For statistical analysis of PUFAs and lipid mediators, nonparametric Wilcoxon signed-rank tests and Mann-Whitney *U* tests were used. Data are expressed as median with minimum and maximum range. Statistical significance is indicated in the figure legends.

## RESULTS

### Atgl^−/−^ mice exhibit Jordans’ anomaly in neutrophil granulocytes

**Figure 1.**
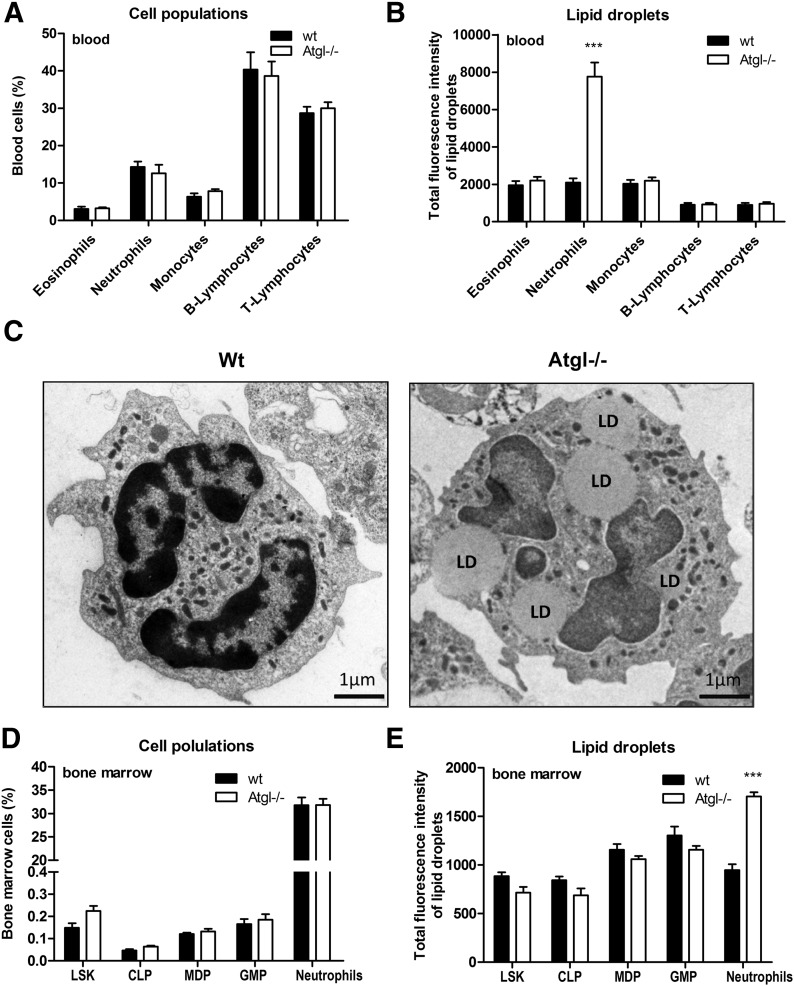
Atgl^−/−^ mice accumulate LDs in neutrophils of peripheral blood and bone marrow. (A) Relative counts of peripheral blood from WT and Atgl^−/−^ mice were determined by immunophenotyping by use of flow cytometry. (B) LDs in peripheral blood cells were quantified by use of BODIPY 493/503 as a neutral lipid stain. WT and Atgl^−/−^ bone marrow were analyzed by transmission electron microscopy (C) and flow cytometry (D and E). LSK, hematopoietic precursors. Data are shown as geometric means of fluorescence intensity + sem (*n* = 5). ***P ≤ 0.001.

Immunophenotyping of peripheral blood by flow cytometry revealed comparable blood counts between WT and Atgl^−/−^ mice (**Fig. 1A**). By use of BODIPY 493/503 as a neutral lipid dye, we observed increased fluorescence intensity, exclusively in neutrophils of Atgl^−/−^ mice (Fig. 1B). To gain insight into the ultrastructural morphology of the cells, we performed transmission electron microscopy of WT and Atgl^−/−^ bone marrow cells. As shown in Fig. 1C, intracellular LDs were present in the cytoplasm of Atgl^−/−^ bone marrow cells as electron-lucent, round-shaped structures, whereas no LDs were visible in WT cells. Next, we evaluated by flow cytometry whether hematopoietic precursor cells of Atgl^−/−^ mice accumulate LDs. Relative populations of bone marrow cells were comparable between WT and Atgl^−/−^ mice (Fig. 1D). We observed an increased amount of LDs exclusively in mature Gr-1^+^ neutrophils of Atgl^−/−^ mice but not in hematopoietic stem cells, such as LSK, or any of the investigated precursor cells, such as CLPs, MDPs, and GMPs (Fig. 1E).

### Pronounced accumulation of LDs under inflammatory conditions in the absence of ATGL

**Figure 2.**
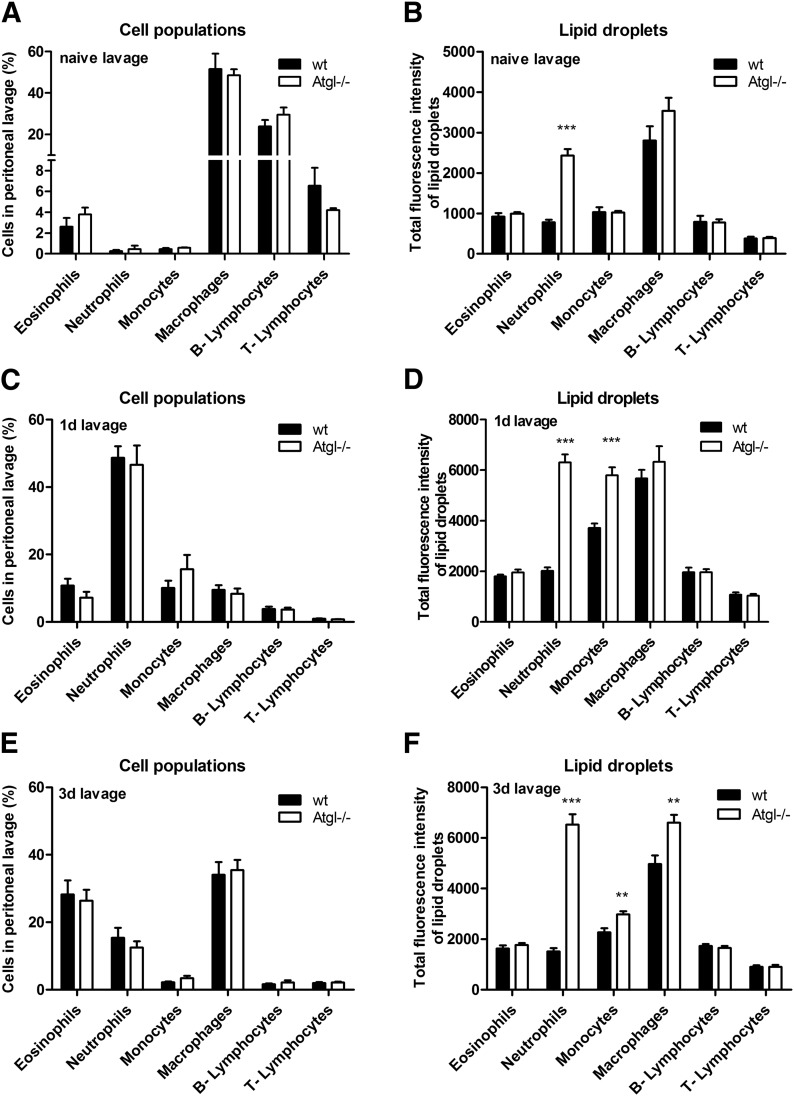
Pronounced accumulation of LDs in Atgl^−/−^ cells under inflammatory conditions. Peritoneal lavage of naive (A and B), 1 d (C and D), and 3 d (E and F) thioglycolate-injected WT and Atgl^−/−^ mice was collected and immunophenotyped by use of BODIPY 493/503 as a neutral lipid stain. Data are shown as geometric means of fluorescence intensity + sem (*n* = 5). **P ≤ 0.01; ***P ≤ 0.001.

**Figure 3.**
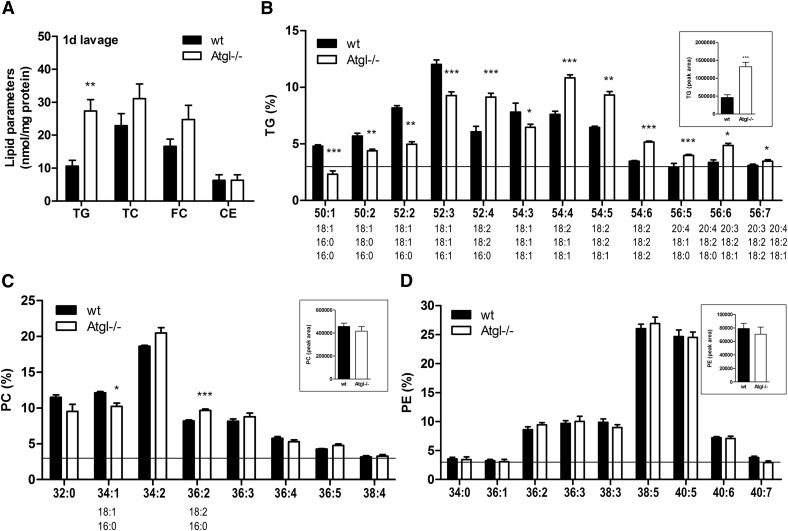
Atgl^−/−^ neutrophils accumulate TG-associated 18:1, 18:2, and 20:4. Lipids of peritoneal lavage cells, 1 d post-thioglycolate injection, were extracted, and concentrations of TGs, TCs, FC, and CEs were determined by enzymatic assays. Data are shown as means + sem (*n* = 5). FA composition of the most abundant TG (B), PC (C), and phosphatidylethanolamine (PE) (D) species (abundance >3%) was determined by LC/ESI-MS. Data are presented as means + sem of absolute (insets) and relative values (*n* = 4–5). *P < 0.05; **P ≤ 0.01; ***P ≤ 0.001.

In naive lavages, resident peritoneal cells include predominantly macrophages, lymphocytes, and small numbers of granulocytes and monocytes (**Fig. 2A**). Similar to our findings in peripheral blood, we observed a selective LD accumulation in neutrophils of naive lavages from Atgl^−/−^ mice (Fig. 2B). To elucidate the effect of ATGL^−/−^ under inflammation-driven conditions, we used a thioglycolate-induced peritonitis model [27]. The cellular infiltration into the peritoneum was unchanged between WT and Atgl^−/−^ mice, 1 d (Fig. 2C) and 3 d (Fig. 2E) postinjection. An increased LD abundance in neutrophils and monocytes was observed on d 1 (Fig. 2D) and in neutrophils, monocytes, and macrophages on d 3 postinjection (Fig. 2F) in peritoneal lavages from Atgl^−/−^ mice. Oil Red O staining of sedimented peritoneal cells confirmed this increased abundance of LDs in cells from Atgl^−/−^ mice, whereas in WT cells, only few LDs were visible (data not shown). Lipid parameters of peritoneal cells isolated from mice 1 d after thioglycolate injection showed a 2.6-fold TG accumulation in Atgl^−/−^ cells. TC, FC, and CE concentrations were unaffected (**Fig. 3A**). With the use of LC-ESI/MS analysis, we found that the relative FA composition of the most abundant TG species (abundance >3% of total TGs), ranging from C_50_ to C_56_, differed significantly between WT and Atgl^−/−^ cells (Fig. 3B). In neutrophils from Atgl^−/−^ mice, TG species with >4 double bonds, including predominantly 18:1, 18:2, 20:3, and 20:4, showed an increased relative abundance, whereas TG species with up to 3 double bonds, containing mostly 16:0, 18:0, and 18:1, were decreased. Absolute concentrations of the most abundant phospholipid species, PC, were unchanged (Fig. 3C, inset). The relative PC composition was changed to an increase in 36:2 and a reduction in 34:1 in Atgl^−/−^ neutrophils (Fig. 3C). No alterations were observed in phosphatidylethanolamine between WT and Atgl^−/−^ neutrophils, neither in absolute (Fig. 3D, inset) nor relative amounts (Fig. 3D).

### Inflammatory cells express the full cascade of metabolic lipases

**Figure 4.**
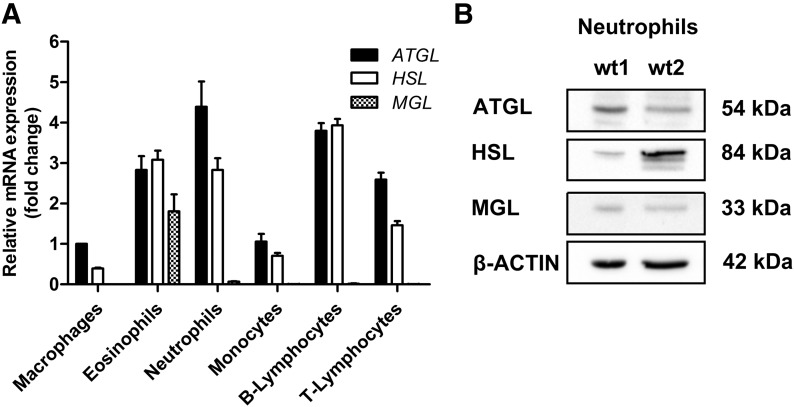
Inflammatory cells express metabolic lipases. (A) Total RNA of flow-sorted inflammatory cells was isolated and analyzed for mRNA expression of *ATGL*, *HSL*, and *MGL* by quantitative real-time PCR. For data normalization, *GAPDH* was used as reference gene. Transcript expression levels are shown as fold change, relative to *ATGL* mRNA levels in macrophages, and represent means + sem (*n* = 4). (B) Protein levels of lipases in isolated neutrophils were analyzed by Western blotting. Total protein lysate (50 μg) from isolated neutrophils/lane was separated by 10% SDS-PAGE under reducing conditions, blotted onto nitrocellulose membrane, and probed with specific antibodies against ATGL, HSL, and MGL. β-Actin was used as a loading control.

Next, we investigated whether lipolytic enzymes are expressed in immune cells. We analyzed flow-sorted macrophages, eosinophils, neutrophils, monocytes (derived from thioglycolate-elicited lavage), as well as B and T lymphocytes (derived from blood) for mRNA expression of *ATGL*, *HSL*, and *MGL* (**Fig. 4A**) by quantitative real-time PCR. mRNA expression of *ATGL* was highest in neutrophils, followed by B lymphocytes, eosinophils, T lymphocytes, monocytes, and macrophages. *HSL* was highly expressed in B lymphocytes and in comparable amounts in eosinophils and neutrophils. T lymphocytes, monocytes, and macrophages showed the lowest levels of *HSL* mRNA. *MGL* was most abundant in eosinophils, whereas all other cell types showed little expression. We confirmed the presence of ATGL (54 kDa), HSL (84 kDa), and MGL (33 kDa) in lysates of purified WT neutrophils via immunoblotting (Fig. 4B). Together, our data demonstrate that metabolic lipases are expressed in inflammatory cells.

### Agonist-induced Ca^2+^ flux and chemotaxis are increased in Atgl^−/−^ neutrophils

**Figure 5.**
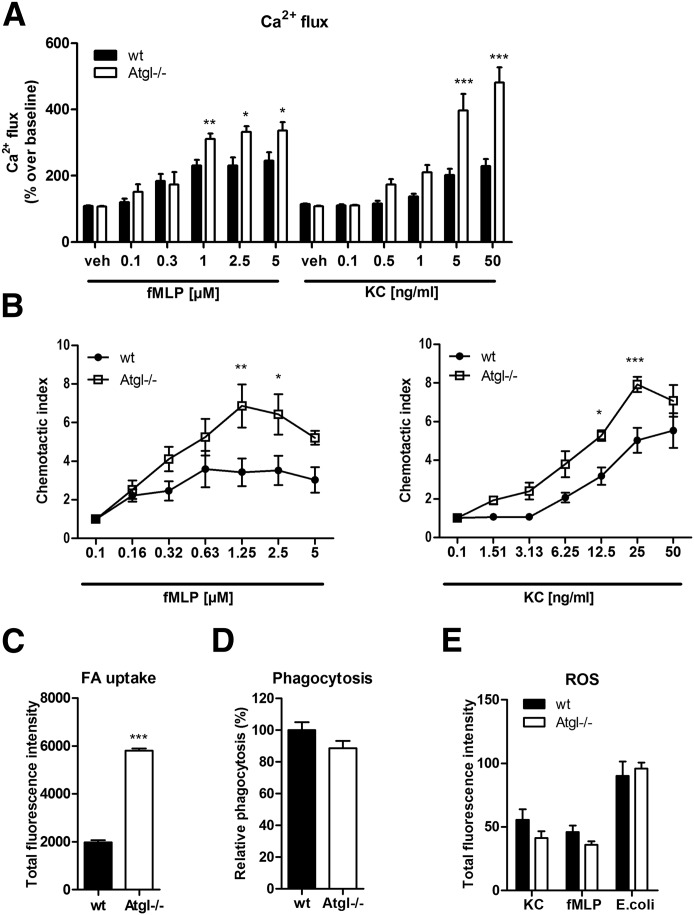
Ca^2+^ flux and chemotaxis are increased in Atgl^−/−^ neutrophils. Activity of neutrophils from WT and Atgl^−/−^ mice was tested in vitro by Ca^2+^ flux (A) and chemotaxis assays (B) in response to fMLP and KC. Data are shown as fold change relative to vehicle-treated control cells ± sem (*n* = 5). (C) Uptake of BODIPY-labeled FAs by WT and Atgl^−/−^ neutrophils. Total fluorescence intensities were measured by flow cytometry and are expressed as means + sem (*n* = 6). (D) Phagocytosis of fluorescein-conjugated *E. coli* particles by WT and Atgl^−/−^ neutrophils. Phagocytosis of WT cells was arbitrarily set to 100%. Data are presented as mean values (*n* = 9–10) + sem. (E) ROS staining after incubation of neutrophils for 20 min with KC (20 ng/ml), fMLP (1 µM), and during 1 h exposure to *E. coli* particles. Data are shown as total fluorescence intensity (*n* = 5). *P < 0.05; **P ≤ 0.01; ***P ≤ 0.001.

Of particular interest was to examine the functionality of LD-rich Atgl^−/−^ neutrophils. Therefore, we determined agonist-induced, [Ca^2+^]_i_ flux and chemotaxis. As agonists, we used the bacterial peptide fMLP and the neutrophil-specific chemoattractant KC (also known as growth-related oncogene α or CXCL1). Compared with WT cells, which reached a maximal Ca^2+^ response of ∼230% over baseline, we observed an enhanced mobilization of [Ca^2+^]_i_ by >300% in Atgl^−/−^ neutrophils with fMLP concentrations ranging from 1 to 5 µM (**Fig. 5A**). KC (5 and 50 µM) induced an even higher response in Atgl^−/−^ cells with peak values of 369% and 447% over baseline in WT and Atgl^−/−^ neutrophils, respectively (Fig. 5A). We next examined the cellular phenotype of Atgl^−/−^ cells by measuring in vitro migration. These studies revealed that neutrophils from Atgl^−/−^ mice possessed increased chemotactic properties toward both tested agonists compared with WT neutrophils (Fig. 5B). As another marker for neutrophil activation, we determined agonist-induced CD11b expression by flow cytometry. fMLP- and KC-mediated stimulation showed a slight yet nonsignificant trend for an increased activation in Atgl^−/−^ neutrophils (Supplemental Fig. 1). By use of PMA to achieve a maximal and robust activation of neutrophils, we found an increased responsiveness in Atgl^−/−^ compared with WT cells.

The intracellular TG accumulation in Atgl^−/−^ neutrophils might be caused by alterations in cellular uptake. After 10 min exposure to fluorescently labeled FAs, neutrophils from Atgl^−/−^ mice showed 2.9-fold increased uptake of FAs compared with control cells (Fig. 5C). Next, we addressed whether the lack of ATGL affects phagocytosis and ROS production of neutrophils. In vitro phagocytosis assays revealed comparable phagocytosis rates between neutrophils from WT and Atgl^−/−^ mice (Fig. 5D). Simultaneously, we measured intracellular ROS formation following synchronized phagocytosis, as well as ROS production, in response to KC and fMLP. All conditions tested showed comparable fluorescence intensities, indicating unchanged ROS concentrations (Fig. 5E). Moreover, we assessed apoptosis under various conditions, which was unaltered between WT and Atgl^−/−^ neutrophils, as Annexin V/propidium iodide staining revealed the same amount of alive, early, and late apoptotic, as well as necrotic, cells (Supplemental Fig. 2).

### Similar but less pronounced phenotype in myeloid-specific Atgl^−/−^ mice

**Figure 6.**
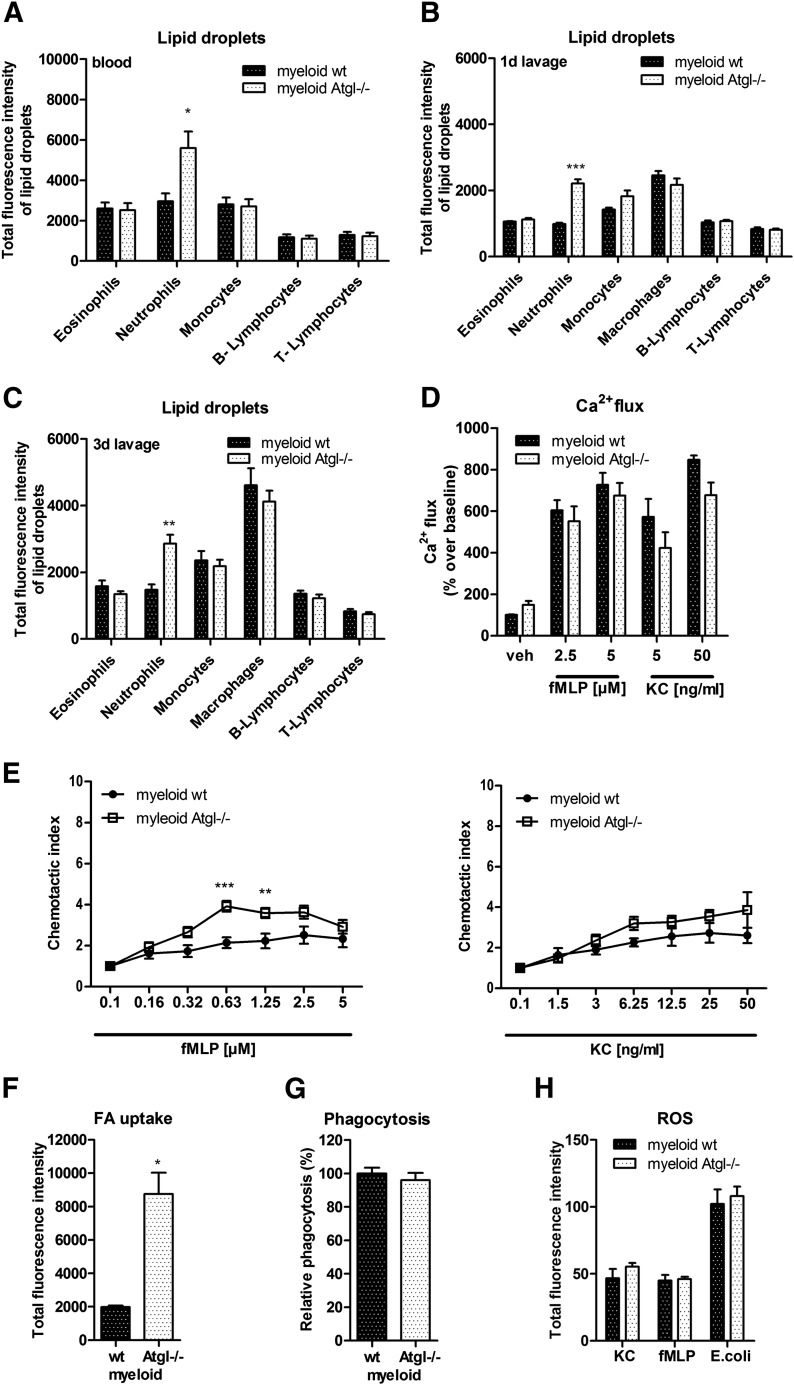
Less pronounced phenotype in neutrophils from myeloid-specific Atgl^−/−^ mice. Cellular LD content in peripheral blood (A) and 1 d (B) and 3 d (C) thioglycolate-elicited peritoneal lavage was quantified by use of BODIPY 493/503. Ca^2+^ flux (D) and chemotaxis assays (E) in response to fMLP and KC. Data are shown as fold change relative to vehicle-treated control cells ± sem (*n* = 5). FA uptake (F), phagocytosis (G), and ROS (H) measurements were performed as described above (*n* = 5). *P < 0.05; **P ≤ 0.01; ***P ≤ 0.001.

To elucidate whether Jordans’ anomaly is caused by the specific lack of ATGL in immune cells or by the systemic lack of ATGL, we included mice with a targeted deletion in myeloid cells. Similar to our observations from global Atgl^−/−^ mice with a 3.7-fold increase in LD abundance in peripheral blood neutrophils, myeloid-specific Atgl^−/−^ mice showed a 1.7-fold increase in neutrophil LDs (**Fig. 6A**). Under inflammatory conditions, myeloid-specific Atgl^−/−^ mice showed an increased LD abundance exclusively in neutrophils, both 1 d (Fig. 6B) and 3 d (Fig. 6C) post-thioglycolate injection, which was of lower magnitude compared with global Atgl^−/−^ mice (2.2-fold in myeloid Atgl^−/−^ vs. 3.1-fold in global Atgl^−/−^ mice). Ca^2+^ flux assays with neutrophils isolated from myeloid-specific Atgl^−/−^ mice that uses fMLP and KC as agonists showed a comparable response as WT cells (Fig. 6D). Chemotaxis was increased in response to fMLP but compared with WT cells after KC stimulation (Fig. 6E). Comparable with neutrophils from global Atgl^−/−^, we found increased FA uptake (Fig. 6F) and no alterations in phagocytosis (Fig. 6G) and ROS formation (Fig. 6H). Together, these observations indicate a similar yet less pronounced phenotype in neutrophils from myeloid-specific versus global Atgl^−/−^ mice.

### Pharmacological inhibition of ATGL results in LD accumulation and reduced release of lipid mediators from neutrophils

**Figure 7.**
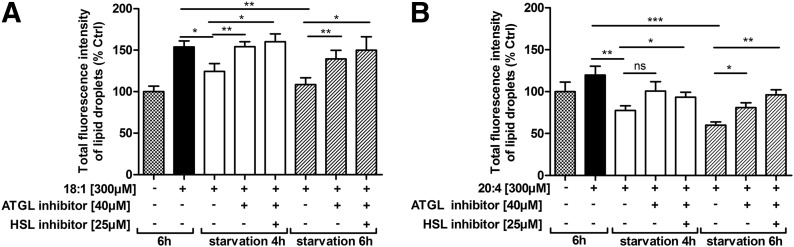
Neutrophil LD catabolism is blocked by the ATGL inhibitor Atglistatin. WT neutrophils collected from peritoneal lavages, 1 d post-thioglycolate injection were incubated in culture medium supplemented with 300 µM OA (18:1) (A) or AA (20:4) (B) for 6 h, after which, the medium was replaced by serum-free DMEM containing ATGL or ATGL/HSL inhibitors. After 4 and 6 h of starvation, LDs were quantified by flow cytometry. Fluorescence intensities are expressed as percentages of untreated control cells + sem (*n* = 6-7). *P < 0.05; **P ≤ 0.01; ***P ≤ 0.001.

To exclude potential systemic effects caused by ATGL^−/−^, we inhibited ATGL in neutrophils ex vivo by treatment with the specific inhibitor Atglistatin [28]. By use of thioglycolate-elicited neutrophils collected from WT mice 1 d after thioglycolate injection, we determined whether Atglistatin effectively inhibited ATGL in these cells. To induce LD formation, we loaded WT neutrophils with 300 µM OA (18:1), and thereafter, we cultured cells in serum-free medium in the absence and presence of Atglistatin. After 4 and 6 h of starvation, we quantified LDs and observed an increased LD content in the presence of Atglistatin (**Fig. 7A**). These results indicate that the inhibitor blocks FA mobilization, which consequently results in TG accumulation. The combined use of Atglistatin and Hi tended to promote accumulation further of LDs in neutrophils. Similar results were observed after loading neutrophils with 300 µM AA (20:4; Fig. 7B).

**Figure 8.**
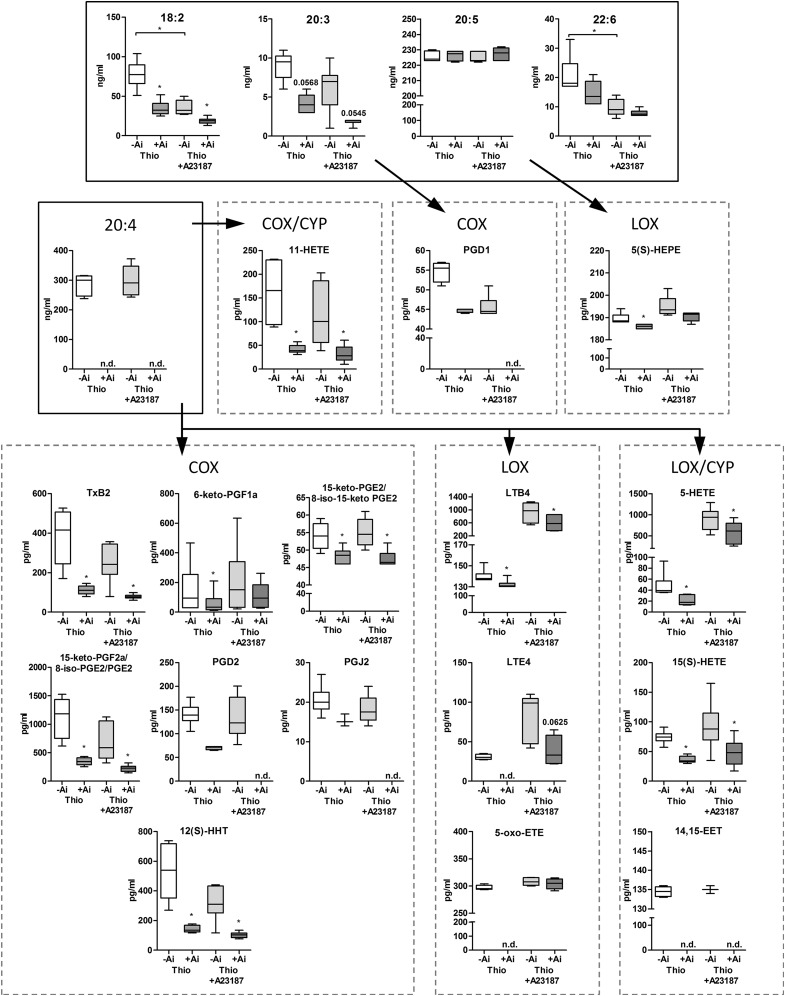
Reduced lipid mediator release upon pharmacological ATGL inhibition. Neutrophils were cultured for 6 h in medium in the absence or presence of Atglistatin. Media were collected from thioglycolate-elicited neutrophils and activated neutrophils (0.5 µM A23187, 1 h). PUFA and lipid mediator concentrations were measured by LC-MS/MS analysis and are presented as median and range (*n* = 6). Ai, Atglistatin; n.d., not detectable; Thio, thioglycolate. *P < 0.05.

Next, we studied the release of PUFAs and lipid mediators from thioglycolate-elicited WT neutrophils after Atglistatin-mediated ATGL inhibition. Atglistatin treatment resulted in reduced release of 18:2 and 20:3 by 58%; 22:6 trended to be decreased, whereas 20:5 levels were unchanged (**Fig. 8**; thioglycolate −Atglistatin vs. +Atglistatin). Concentrations of 20:4 were below the detection limit after ATGL inhibition. As shown in Supplemental Table 1, detected lipid mediators, derived from all 3 major enzymatic pathways—COX, LOX, and CYP—were present in the media after 1 h ex vivo culture of thioglycolate-elicited peritoneal neutrophils. Upon ATGL inhibition, predominantly 20:4-derived metabolites were less abundant compared with media from cells without inhibitor (Fig; 8 and Supplemental Table 1). Reduced metabolites included the COX-derived TXB_2_, PGD_2_, PGE_2_, and PGJ_2_, 6-keto-PGF_1a_, and 12(S)-HHT, the LOX-derived LTB_4_, LTE_4_, and 5-oxo-ETE, the COX/CYP-derived 11-HETE, and the LOX/CYP-derived 15(S)-HETE, 5-HETE, and 14,15-EET. The effect of ATGL inhibition was not restricted to eicosanoids arising from 20:4. We also observed significantly reduced levels of the 20:3-derived PGD_1_ and 20:5-derived 5(S)-HEPE lipid mediators.

In addition, we investigated lipid mediator release from thioglycolate-elicited neutrophils after ATGL inhibition when exposed to the Ca^2+^ ionophore A23187 as additional stimulus. When compared with thioglycolate-only-stimulated conditions, neutrophil activation by A23187 was associated with reduced concentrations of 18:2 and 22:6 in the medium, whereas levels of 20:3, 20:4, and 20:5 released from thioglycolate- and A23187-stimulated cells were comparable (Fig. 8; −Atglistatin: thioglycolate vs. thioglycolate + A23187). Ca^2+^ ionophore treatment resulted in increased release of LOX-derived lipid mediators, in particular, LTE_4_ (3-fold), LTB_4_ (7-fold), and 5-HETE (20-fold) when compared with thioglycolate-stimulated WT neutrophils (Fig. 8 and Supplemental Table 1; −Atglistatin: thioglycolate vs. thioglycolate + A23187). This observation is in line with previous reports describing A23187 as a stimulus predominantly targeting the 5-LOX pathway [29]. ATGL inhibition showed a similar reduction in lipid mediator release in A23187- and thioglycolate-only-stimulated cells (Fig. 8; thioglycolate + A23187: −/+Atglistatin).

### Reduced concentrations of lipid mediators in peritoneal exudates from Atgl^−/−^ mice

**Figure 9.**
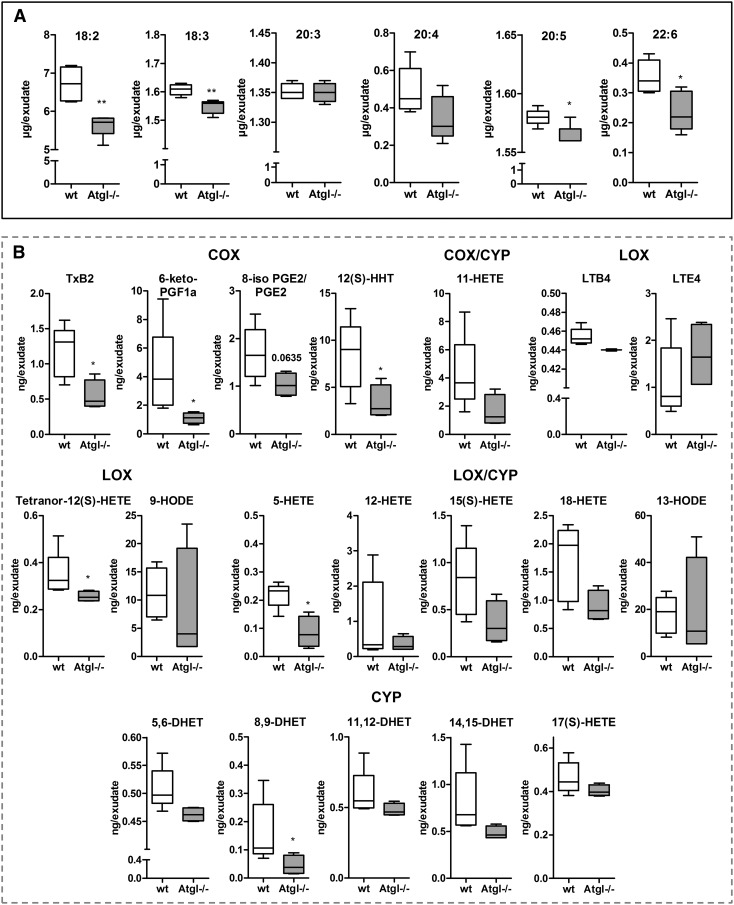
Reduced lipid mediator concentrations in peritoneal lavages from Atgl^−/−^ mice. Cell-free fluids of peritoneal lavages from WT and Atgl^−/−^ mice, 1 d post-thioglycolate injection, were collected. PUFA (A) and lipid mediator (B) concentrations were measured by LC-MS/MS. HODE, hydroperoxyoctadecadienoic acid. Data are shown as median and range (*n* = 4–5). *P < 0.05, **P ≤ 0.01.

To substantiate our in vitro findings and to test the in vivo impact of ATGL^−/−^, we examined cell-free lavage fluids of 1 d thioglycolate-elicited peritoneal lavages from WT and global Atgl^−/−^ mice with respect to lipid mediator concentrations. PUFAs in the lavage fluids of global Atgl^−/−^ mice showed reduced concentrations of 18:2, 18:3, 20:5, and 22:6, whereas 20:3 and 20:4 levels were comparable between WT and Atgl^−/−^ lavages (**Fig. 9**). The lipid mediator profile revealed the presence of metabolites arising from all 3 major enzymatic pathways. The amount of the COX-derived TXB_2_ (the stable metabolite of TXA_2_), 6-keto-PGF_1a_, and 12(S)-HHT, LOX-derived tetranor-12(S)-HETE and 5-HETE, and CYP-derived 8,9-DHET was reduced significantly in peritoneal exudates from Atgl^−/−^ compared with WT mice. Among these, TXB_2_ and 5-HETE are mediators typically released from neutrophils under inflammatory conditions. Several detectable mediators, such as LTB_4_ and PGE_2_, various HETEs [11-HETE, 15(S)-HETE, 18-HETE], and DHETs, the diol metabolites of EETs, showed a trend for reduced levels, which, however, did not reach statistical significance.

## DISCUSSION

Lipolysis is mainly catalyzed by ATGL, which specifically cleaves TG in cytosolic LDs. We show here that the systemic loss of ATGL in mice results in an increased abundance of TG-rich LDs specifically in neutrophil granulocytes from bone marrow, peripheral blood, and peritoneal lavage under basal conditions. Under inflammatory conditions, we observed that thioglycolate-elicited inflammation triggers LD formation in all investigated immune cells from WT and Atgl^−/−^ mice. This is evident by an increased amount of LDs in cells from thioglycolate-injected compared with naive lavages. Glycosylated proteins in the thioglycolate medium activate receptors for advanced glycation end products, which further induce proinflammatory responses through NF-κB-mediated secretion of cytokines and inflammatory factors [30]. In Atgl^−/−^ cells, this proinflammatory stimulus boosts LD accumulation to increased amounts of LDs in neutrophils, monocytes, and macrophages, whereas eosinophils and B and T lymphocytes do not show any alterations in LD content between WT and Atgl^−/−^ mice. This finding is somehow unexpected, as all immune cells investigated express ATGL. However, as inflammation-mediated induction of LDs in leukocytes is highly cell and stimulus dependent [31], it remains to be determined whether other stimuli are required to trigger ATGL-mediated functions in these cells.

LDs have a dual function within cells by controlling lipid storage, thereby protecting cells from lipotoxicity and by serving as energy fuel upon starvation. In contrast to many other cell types, in which ATGL-mediated lipolysis represents an important biochemical mechanism to generate FAs as energy substrates, most lymphoid and myeloid cells (including neutrophils) derive their energy exclusively from glycolysis [32]. In accordance, we did not observe alterations in cell viability or any limitations in highly energy-demanding processes, such as migration and phagocytosis in Atgl^−/−^ neutrophils. Of note, the absence of ATGL was associated with an elevated Ca^2+^ mobilization and increased chemotaxis of neutrophils toward chemoattractants, indicating a proinflammatory phenotype of these cells. This phenotype was more pronounced in cells from global compared with myeloid-specific Atgl^−/−^ mice, indicating that these alterations are likely caused by secondary effects as a result of systemic ATGL^−/−^ rather than by the cell-specific lack of ATGL. A causative reason for this might be additional factors provoked by the severe phenotype of global ATGL^−/−^, including perivascular oxidative stress and endothelial dysfunction [33], ER stress, and mitochondrial dysfunction in various tissues [34], with the most dramatic phenotype in cardiac muscle resulting in lethal cardiomyopathy within a few months after birth [35].

cPLA_2α_ (also known as group IVA PLA_2_) is assigned a central role in stimulus-dependent 20:4 mobilization from phospholipids during eicosanoid generation. Subcellular eicosanoid-generating compartments include membranes of the ER, phagosome, nuclear envelope, and LD but not the plasma membrane [17]. In a recent study, the TG-rich core of LDs in human mast cells has been identified as an additional substrate reservoir for eicosanoid precursors, with ATGL being critically involved in the release of these precursor FAs [36]. Our observations further support and extend this study showing that ATGL inhibition leads to an accumulation of TG-rich cytoplasmic LDs in neutrophils, monocytes, and macrophages and results in a reduced production of lipid mediators from neutrophils. A central question arising from the mast cell study was whether ATGL provides substrates for lipid mediator synthesis by its TG hydrolase activity [37] or its putative phospholipase activity [38]. Furthermore, it remained to be determined whether ATGL-mediated 20:4 release provides substrates directly for eicosanoid generation or indirectly by replenishing 20:4 in glycerophospholipids for the subsequent release by cPLA_2α_. Our lipidomics data clearly show a pronounced increase of 18:1, 18:2, and 20:4 in the TG fraction in the absence of ATGL and no accumulation of linoleyl- or arachidonyl-containing PC or phosphatidylethanolamine, demonstrating that ATGL acts as a TG hydrolase and directly liberates FAs. FA mobilization and release are critical processes, and Atgl^−/−^ neutrophils try to compensate for defective lipolysis by increased FA uptake from extracellular sources.

Genetic and pharmacological ATGL inhibition, both in vivo and in vitro, reduces the release of some but not all lipid mediators derived from all 3 enzymatic pathways. Lipid mediator generation is regulated in a highly time-dependent manner at various levels, e.g., through the availability of enzymes and substrates, as well as the site of mediator generation within different cellular compartments [17]. Our results indicate that lipid mediators, which remain unaffected by ATGL inhibition, are derived from non-LD compartments, whereas mediators with a reduced release upon ATGL inhibition are specifically generated from the TG-LD pool in an ATGL-dependent manner. Recently, a comprehensive work describing the cellular and molecular events, including lipid mediator generation in mice with thioglycolate-induced peritonitis, has been published [27]. This study revealed that in this model, the inflammatory response after 24 h is characterized by high levels of 20:4-derived proinflammatory eicosanoids, such as PGD_2_, LTB_4_, PGE_2_, 5-HETE, and TXB_2_. This time point correlates with a high infiltration of the peritoneal cavity with neutrophils, rendering 24 h as a suitable time point to investigate neutrophil-mediated lipid mediator release. In accordance, we detected a release of these eicosanoids from lavages collected from 1 d thioglycolate-stimulated mice, which were reduced in Atgl^−/−^ mice. As eicosanoids are critical determinants for the modulation of an inflammatory response, a dysregulation in any dimension might have pathologic consequences, such as chronic inflammation, failure of pathogen clearance, and both local and distal tissue damage [39, 40]. The physiologic significance of ATGL^−/−^ associated with reduced eicosanoid generation remains to be determined.

Clinical reports from patients with mutations in the ATGL gene describe an analogous phenotype as Atgl^−/−^ mice: they suffer from massive TG accumulation in multiple tissues and develop severe myopathy, which often requires cardiac transplantation [41]. In humans, Jordans’ anomaly affects various immune cells, predominantly granulocytes [42, 43]. In some case reports of NLSDM, an increased prevalence of reoccurring infections was observed [44]. Unfortunately, it is not elusive whether these immune defects can be linked to Jordans’ anomaly and whether eicosanoid production by blood leukocytes is altered in these patients. In general, a direct correlation between LD number and eicosanoid release was described in human and murine leukocytes [45]. Thus, the increased presence of LDs in neutrophils from humans and mice with Jordans’ anomaly should result in an increased potential of these cells to generate lipid mediators. As a result of inhibited lipid mobilization from LDs in Atgl^−/−^ conditions, however, the release of lipid mediators is partly inhibited, further underlining the important role of ATGL in the regulation of eicosanoid generation. Observations that mutations in the ATGL coactivator CGI-58 also result in Jordans’ anomaly in humans raise the possibility of a similar phenotype in immune cells deficient in CGI-58. Macrophages lacking ATGL or CGI-58, however, showed opposing phenotypes in terms of macrophage function and atherosclerosis susceptibility [46], rendering expectations in this context rather speculative.

In summary, our work strengthens the hypothesis that “lipolysis meets inflammation” with ATGL and probably other lipid hydrolases participating in inflammatory signaling processes [47]. Our data provide mechanistic insights that the release of lipid mediators from neutrophils is dependent on liberation of FAs as precursor molecules from the TG-rich pool of LDs by ATGL. As lipid mediator-synthesizing LDs are not only restricted to neutrophils but are also present in many other immunomodulatory, endothelial, epithelial, and neoplastic cells, it is reasonable to speculate that ATGL regulates FA availability and consequently, the release of lipid mediators also in these cells. Our work provides an important basis for future experiments exploring the role of ATGL-mediated lipid mediator release in the context of inflammatory and neoplastic diseases.

## AUTHORSHIP

S.S. and D. Kratky conceived of the study and wrote the manuscript. S.S., D. Kratky, H.S., and U.C. designed the research. S.S., M.G., N.V., K.J., R.F., D. Kolb, J.D., T.O.E., and A.R. performed experiments. E.E.K. provided myeloid-specific Atgl^−/−^ mice. S.S., A.W., and J.D. analyzed data. A.L., A.D., and A.H. critically revised the manuscript.

## Abbreviations

5-oxo-ETE: 5-oxo-eicosatetraenoic acid
12(S) HHT: 12(S)-hydroxy-5,8,10 (Z,E,E)-heptadecatrienoic acid
AA: arachidonic acid
APC: allophycocyanin
ATGL: adipose triglyceride lipase
ATGL^−/−^: adipose triglyceride lipase deficiency
[Ca^2+^]_i_: intracellular Ca^2+^
CE: cholesterol ester
CGI-58: comparative gene identification-58
CLP: common lymphoid progenitor
COX: cyclooxygenase
cPLA_2α_: cytosolic phospholipase A_2α_
CYP: cytochrome P450
DHET: dihydroxyeicosatrienoic acid
EET: epoxyeicosatrienoic acid
ER: endoplasmic reticulum
ESI: electrospray ionization
FA: fatty acid
FC: free cholesterol
GMP: granulocyte/monocyte progenitor
HEPE: hydroxyeicosapentaenoic acid
HETE: hydroxyeicosatetraenoic acid
Hi: hormone-sensitive lipase inhibitor Hi 76-0079
HSL: hormone-sensitive lipase
KC: keratinocyte-derived chemoattractant
LC: liquid chromatography
LD: lipid droplet
LOX: lipoxygenase
LSK: Lin^−^Sca-1^+^cKit^+^
LTB/E_4_: leukotriene B/E_4_
MDP: monocyte/dendritic cell progenitor
MGL: monoacylglycerol lipase
MS: mass spectrometry
NLSD: neutral lipid storage disease
NLSDM: neutral lipid storage disease with myopathy
OA: oleic acid
PC: phosphatidylcholine
PLA_2_: phospholipase A_2_
PNPLA2: patatin-like phospholipase domain containing 2
PUFA: polyunsaturated fatty acid
ROS: reactive oxygen species
TC: total cholesterol
TG: triglyceride
TXA/B_2_: thromboxane A/B_2_
WT: wild-type
